# GPCRomics of Homeostatic and Disease-Associated Human Microglia

**DOI:** 10.3389/fimmu.2021.674189

**Published:** 2021-05-14

**Authors:** Cheng-Chih Hsiao, Roman Sankowski, Marco Prinz, Joost Smolders, Inge Huitinga, Jörg Hamann

**Affiliations:** ^1^ Department of Experimental Immunology, Amsterdam institute for Infection and Immunity, Amsterdam University Medical Centers, Amsterdam, Netherlands; ^2^ Neuroimmunology Research Group, Netherlands Institute for Neuroscience, Amsterdam, Netherlands; ^3^ Institute of Neuropathology, Faculty of Medicine, University of Freiburg, Freiburg, Germany; ^4^ Signalling Research Centres BIOSS and CIBSS, University of Freiburg, Freiburg, Germany; ^5^ Center for Basics in NeuroModulation (NeuroModulBasics), Faculty of Medicine, University of Freiburg, Freiburg, Germany; ^6^ MS Center ErasMS, Departments of Neurology and Immunology, Erasmus Medical Center, Rotterdam, Netherlands; ^7^ Swammerdam Institute for Life Sciences, Center for Neuroscience, University of Amsterdam, Amsterdam, Netherlands

**Keywords:** brain, microglia, GPCRs, G proteins, system biology, multiple sclerosis, CXCR4

## Abstract

G-protein-coupled receptors (GPCRs) are critical sensors affecting the state of eukaryotic cells. To get systematic insight into the GPCRome of microglia, we analyzed publicly available RNA-sequencing data of bulk and single cells obtained from human and mouse brains. We identified 17 rhodopsin and adhesion family GPCRs robustly expressed in microglia from human brains, including the homeostasis-associated genes *CX3CR1*, *GPR34*, *GPR183*, *P2RY12*, *P2RY13*, and *ADGRG1*. Expression of these microglial core genes was lost upon culture of isolated cells *ex vivo* but could be acquired by human induced pluripotent stem cell (iPSC)-derived microglial precursors transplanted into mouse brains. *CXCR4* and *PTGER4* were higher expressed in subcortical white matter compared to cortical grey matter microglia, and *ADGRG1* was downregulated in microglia obtained from normal-appearing white and grey matter tissue of multiple sclerosis (MS) brains. Single-cell RNA sequencing of microglia from active lesions, obtained early during MS, revealed downregulation of homeostasis-associated GPCR genes and upregulation of *CXCR4* expression in a small subset of MS-associated lesional microglia. Functional presence of low levels of CXCR4 on human microglia was confirmed using flow cytometry and transwell migration towards SDF-1. Microglia abundantly expressed the GPCR down-stream signaling mediator genes *GNAI2* (α_i2_), *GNAS* (α_s_), and *GNA13* (α_13_), the latter particularly in white matter. Drugs against several microglia GPCRs are available to target microglia in brain diseases. In conclusion, transcriptome profiling allowed us to identify expression of GPCRs that may contribute to brain (patho)physiology and have diagnostic and therapeutic potential in human microglia.

## Introduction

Microglia are brain-resident phagocytic cells that contribute to brain homeostasis as well as disease (1, 2). Populating the central nervous system (CNS) during embryonic development, microglia persist for the rest of life through local self-renewal. As a consequence, they possess a unique transcriptional signature that emerged only recently from RNA sequencing (RNAseq) of purified primary cells (3). Notably, G protein-coupled receptors (GPCRs) figure prominently in the microglia transcriptome, as exemplified by the characteristic surface expression of CX3CR1, GPR34, GPR183, P2Y_12_, P2Y_13_, and GPR56 (3, 4). GPCRs are the senses of our cells, comprising the largest and most diverse superfamily of membrane proteins in eukaryotic cells. Of particular interest is their widespread cellular distribution and the fact that ∼30% of all currently approved pharmaceuticals target them (5). GPCRs control cell and tissue physiology by regulating signaling pathways *via* heterotrimeric G proteins, which modulate cellular levels of second messengers and, in turn, a wide array of functional activities in all types of cells (6). Microglial GPCRs have been implicated in control of axon outgrowth and cortical laminar positioning during development as well as in support of survival of neurons (CX3CR1), in plasticity (P2Y_12_) and complement-mediated pruning (C3AR1 and C5AR1) of synapses, in microglial brain colonization (CXCR4) and chemotaxis of microglia to injury (P2Y_12_), and in neuropathic pain response (P2Y_12_) (7). Exploring bulk and single cell RNAseq studies of microglia from mice and human, we here describe the expression of GPCR and G protein genes in relation to microglia homeostasis, location, health, and disease.

## Methods

### RNAseq and Microarray Datasets

Genome-wide gene expression data of microglia, macrophages, and non-phagocytic cells were derived from various publicly available RNAseq data sets (**Table 1**). Numbers indicating gene expression are provided either as absolute counts, presenting fragments per kilobase of transcript per million mapped reads (FPKM) or transcripts per kilobase million (TPM), or as relative counts, related to arbitrary chosen, fixed thresholds of all genes in the gene set (e.g., the top 50% = 0.50 percentile). Heatmaps show gene expression intensity, based on the average of all genes, with white indicating low expression and red indicating high expression. *t*-distributed stochastic neighbor embedding (*t-SNE*) plots of the clusters of microglia were generated as described (15). Relative RNA expression levels for *CXCR4* and *CXCL12* from laser-dissected tissue from mixed active/inactive and inactive demyelinated lesions were obtained from a microarray dataset (18).

**Table 1:** RNAseq datasets analyzed in this study.

Species	Cells	Dataset	Reference
various	microglia	bulk RNAseq of isolated cells from cortex (GM) (human) or whole brains (mouse)	Geirsdottir et al. (8)
human	brain lysate, microglia	bulk RNAseq of isolated cells from cortex (GM)	Galatro et al. (9)
human	microglia	bulk RNAseq of isolated cells from cortex (GM)	Gosselin et al. (10)
human	microglia	bulk RNAseq of isolated cells from corpus callosum (WM) and occipital cortex (GM) of control and MS donors	Van der Poel et al. (4)
human	microglia	bulk RNAseq of isolated cells from corpus callosum (WM) and occipital cortex (GM) of control donors	Mizee et al. (unpublished data)
mouse	various	bulk RNAseq of (isolated cells from) whole brains (neurons, oligodendrocytes, astrocytes, and microglia)	Zhang et al. (11)
mouse	microglia, macrophages	bulk RNAseq of isolated cells from whole organs	Van Hove et al. (12)
human	iPSC-derived iMPs	bulk RNAseq of isolated cells transplanted into the brain of neonatal mice in vivo or cultured in vitro	Svoboda et al. (13)
mouse	microglia	single cell RNAseq of cells from brains of 5XFAD mice	Keren-Shaul et al. (14)
human	microglia	single cell RNAseq of cells from cortical (GM) biopsies of active MS lesions	Masuda et al. (15)
human	microglia	single cell RNAseq of cells from cortical (GM) surgically resected brain tissue	Olah et al. (16)
human	microglia	bulk RNAseq of isolated cells from cerebral cortex (GM)	Olah et al. (17)

WM, white matter; GM, grey matter.

### Flow Cytometry

Isolated human microglia from corpus callosum and subcortical white matter and occipital cortex grey matter were isolated and stained as described (4) with APC-conjugated anti-CD11b (clone ICRF44; eBioscience, San Diego, CA, USA), Alexa 700-conjugated anti-CD14 (clone MφP9; BD Biosciences, San Diego, CA, USA), PerCP-Cy5.5-conjugated anti-CD15 (clone HI98; BioLegend, San Diego, CA, USA), BB515-conjugated anti-CD45 (clone HI30; BioLegend), PE-Cy7-conjugated anti-CXCR4 (clone 12G5; BioLegend), and PE-conjugated anti-P2Y_12_ (clone S16007D; BioLegend). Dead cells were visualized by fixable viability dye eFluor 780 (eBioscience). Background staining was determined using fluorescence minus one control. Membrane protein expression was measured on a FACSCanto II (BD Biosciences), and median fluorescence intensity was determined with FlowJo software version 10.1 (Ashland, OR, USA).

### Transwell Migration

Human brain microglia and paired peripheral blood monocytes were separately isolated by CD11b and CD14 microbeads (Miltenyi Biotec, Bergisch Gladbach, Germany) as described (19). 2 x 10^5^ cells were loaded in a volume of 100 μl RPMI1640 medium containing 0.3% bovine serum albumin on transwell filters with a pore size of 5 μm (Corning, Corning, NY, USA). 100 ng/ml SDF-1 (stromal cell-derived factor-1, CXCL12) was added as chemoattractant to the lower compartment. After 4 h incubation at 37°C, cells in the lower compartment were harvested and quantified by flow cytometry at a fixed high speed for 120 sec.

### Statistics

All analyses were performed in GraphPad Prism 7 (GraphPad Software, San Diego, CA, USA). When data were not normally distributed, non-parametric tests, either Wilcoxon or Mann-Whitney U, were performed.

## Results

### Species and Cell Type Specificity of GPCR and GPCR Signaling Molecule Expression in Microglia

To explore the presence of GPCRs in microglia, we utilized the list of GPCRs not involved in olfaction, taste, light perception, and pheromone signaling as provided by the International Union of Basic and Clinical Pharmacology (IUPHAR)/British Pharmacological Society (BPS) Guide to Pharmacology (https://www.guidetopharmacology.org) (20). According to the GRAFS classification (21), the 384 receptors comprise 303 rhodopsin, 33 adhesion, 22 glutamate, 15 secretin, and 11 frizzled family members.

**Figure 1:**
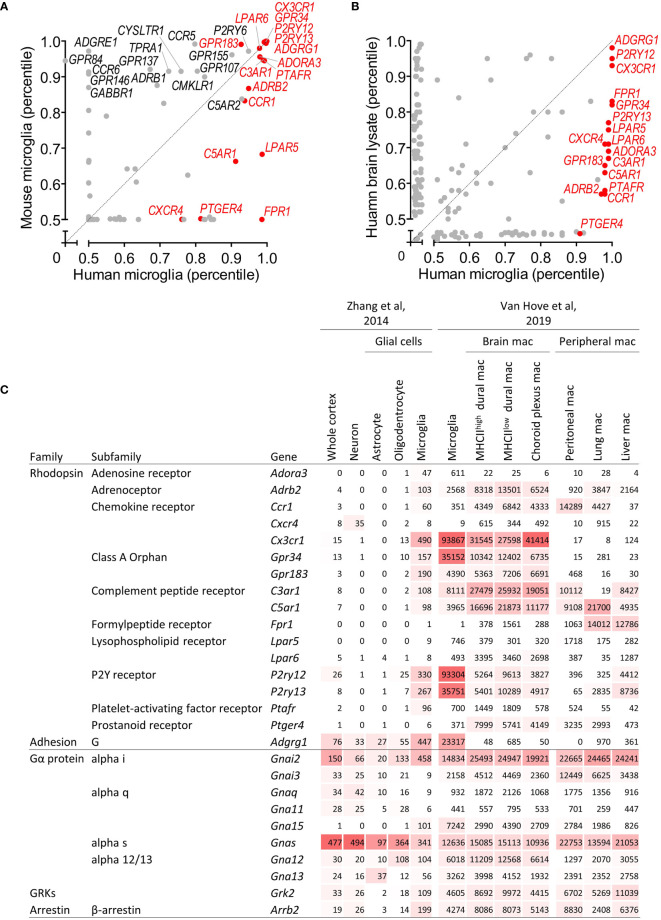
Identification of GPCR and GPCR signaling molecule genes expressed in microglia. **(A)** Scatter plot providing GPCR gene expression in microglia isolated from human and mice as percentile of all human/mouse genes (8). **(B)** Scatter plot providing GPCR gene expression in brain and microglia of human as percentile of all genes (10). **(C)** Expression of GPCR and GPCR signaling molecule genes in mouse cortical tissue, neurons, astrocytes, oligodendrocytes, and microglia (11), and in mouse microglia, non-parenchymal brain and peripheral tissue macrophages (12). Expression is provided as FPKM (11) and gene count (12), respectively. mac, macrophages.

To identify GPCR genes that are reliably expressed in human microglia, we first tested bulk RNAseq expression data of homeostatic microglia from different vertebrate species we recently published (8). We found 83 GPCR genes in the top 50% (0.5 percentile) of all genes in the human and/or mouse gene sets (**Figure 1A**). 42 of these genes were lowly expressed (0.5–0.67 percentile), 12 genes were medium expressed (0.68–0.85 percentile), and 29 genes belonged to the highly expressed genes (0.86–1.0 percentile). While the homeostatic microglia marker genes *CX3CR1*, *GPR34*, *GPR183*, *P2RY12*, *P2RY13*, and *ADGRG1* (encoding GPR56) were highly expressed in both species, expression of other GPCR genes was medium, low, or even absent in either human or mouse. For example, transcription of *ADGRE1*, encoding F4/80 in mouse, was lacking in human microglia, in line with its exclusive expression in human eosinophils (22). Other genes with a restricted, high expression in either mouse or human microglia were *CCR6*, *GPR84*, *GPR146*, and *FPR1*, respectively. Notably, *ADGRE5* (encoding CD97), which is abundantly expressed by all types of bone marrow-derived leukocytes (23) was lowly expressed in mouse and human microglia, in line with previous findings (24). The previously disputed gene *ADGRB1* (encoding BAI1) (25) was undetectable in mouse and human microglia.

We confirmed the list of well-expressed human microglia GPCR genes in four other bulk RNAseq studies of primary human microglia [(4, 9, 10) and Mizee et al, unpublished data]. We found 15 genes abundantly expressed across the different data sets, which formed, together with two genes with a medium expression in grey matter microglia but high expression in white matter microglia (see below), the core microglia GPCR gene set for our further analyses (for all other genes, see the supplementary information). This gene set comprises rhodopsin (e.g., adenosine, chemokine, complement peptide, lysophospholipid, purinergic, and orphan receptors) as well as adhesion, but not secretin, glutamate, or frizzled family members. Among the 17 selected genes were the homeostatic microglia marker genes *CX3CR1*, *GPR34*, *GPR183*, *P2RY12*, *P2RY13*, and *ADGRG1*.

We next tested the expression of microglial GPCRs in other cell types of the CNS. The three most highly expressed genes, *ADGRG1*, *P2RY12*, and *CX3CR1*, also appeared top-abundant in whole brain tissue (10) (**Figure 1B**). All selected genes were more abundantly expressed in pure microglia as compared to the whole human cortex, which was further corroborated by data on gene expression in the major cell types of the mouse CNS (11) (**Figure 1C** and **Supplementary Table 1**). Except for *Cxcr4*, expression in microglia was higher as compared to neurons, astrocytes, and oligodendrocytes. Interestingly, high expression in microglia regularly correlated with gene activity in oligodendrocytes, albeit at a lower level.

Next to parenchymal microglia, the CNS harbors border-associated macrophages with distinct transcriptional signatures, residing in the dura mater, subdural meninges, and choroid plexus (12). Most microglia GPCRs were expressed also in non-parenchymal macrophages of the CNS, at comparable, higher, or lower level (**Figure 1C** and **Supplementary Table 1**). Inclusion of tissue-resident macrophages from peritoneum, lung, and liver unveiled similar patterns (12). The signature genes *Cx3cr1*, *Gpr34*, *P2ry12*, *P2ry13*, and *Adgrg1* were particularly expressed in microglia.

Upon ligation, GPCRs diversify downstream signaling through four main classes of Gα subunit – Gα_s_, Gα_i/o_, Gα_q/11_, and Gα_12/13_ (6). Moreover, G protein-coupled receptor kinases (GRKs) phosphorylate intracellular domains of GPCRs and function together with β-arrestin to regulate the GPCR desensitization (6). Human and mouse microglia expressed *GNAI2* (Gα_i2_), *GNAS* (Gα_s_), *GNA13* (Gα_13_), *GRK2*, and *ARRB2*. In addition, mouse microglia also expressed *Gna15* (Gα_15_) (**Figure 1C** and **Supplementary Table 1**). *Gnai2*, *Gna15*, *Grk2*, and *Arrb2* were higher expressed in microglia as compared to neurons, astrocytes, and oligodendrocytes, while *Gnas* and *Gna12* were expressed in different cell types. G protein, GRK, and β-arrestin gene expression levels in macrophages in- and out-side the CNS were mostly comparable.

### Regional and Disease Specificity of GPCR and GPCR Signaling Molecule Expression in Human Microglia

**Figure 2:**
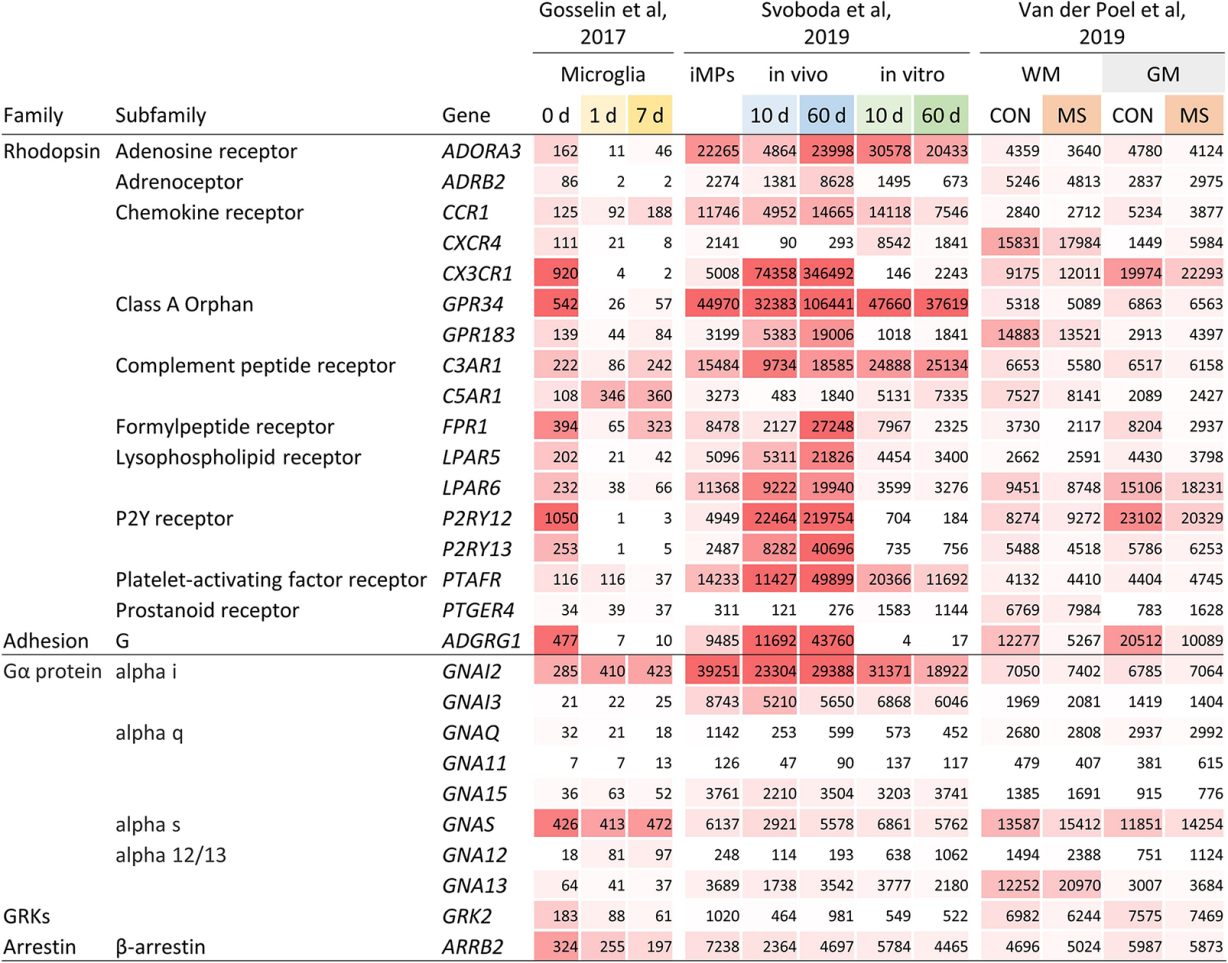
Gene expression of selected GPCRs and GPCR signaling molecules in human microglia. Expression of GPCR and GPCR signaling molecule genes in microglia cultured *ex vivo* for 1 and 7 days (10), in iPSC-derived iMPs transplanted neonatal mouse brains or cultured *in vitro* for 10 and 60 days (13), and in white matter and grey matter microglia from control and MS brains (4). Expression is provided as TPM (10) and gene count (4, 13), respectively. CON, control; WM, white matter; GM, grey matter.

Dissected from their natural microenvironment, microglia change their transcriptional program and alter morphological and functional characteristics (10, 19). Indeed, expression of *ADORA3*, *ADRB2*, *CXCR4*, *CX3CR1*, *GPR34*, *LPAR5*, *LPAR6*, *P2RY12*, *P2RY13*, and *ADGRG1* was strongly downregulated or even lost in microglia cultured *ex vivo* for 1 or 7 days (**Figure 2** and **Supplementary Table 2**).

Human induced pluripotent stem cells (iPSCs) can be differentiated into induced microglial precursors cells (iMPs) showing the characteristic morphology and gene expression of primary human microglia (13). Notably, iMPs expressed the GPCRs typically found in microglia. When transplanted into the brains of NOD scid gamma (NSG) mice, carrying the human transgenes encoding IL-3, SCF, GM-CSF, and CSF1, expression of *ADRB2*, *CX3CR1*, *GPR183*, *FPR1*, *LPAR5*, *LPAR6*, *P2RY12*, *P2RY13*, and *ADGRG1* was further enhanced at day 10 and/or 60 (**Figure 2** and **Supplementary Table 2**). This induction was not seen in iMPs cultured for the same period *in vitro*, which rather resulted in a downregulation of the expression of several GPCRs.

Various studies have established regional differences in microglia gene expression (2). Using data from our laboratory, we compared GPCR expression between human microglia obtained from subcortical white and cortical grey matter (4) (**Figure 2** and **Supplementary Table 2**). We found seven genes ≥2-fold higher expressed in either white or grey matter, respectively. In a second, independent dataset, we could confirm a higher gene expression in white matter microglia for *CXCR4* and *PTGER4* (Mizee et al, unpublished data). Single cell RNAseq has facilitated the investigation of microglial heterogeneity within brain regions. A study addressing this question showed, with the exception of *C3AR1*, a similar activity of GPCR genes in cortical microglia subsets (16) (**Supplementary Figure 1**).

Changes in gene expression in mouse models for Alzheimer’s disease and amyotrophic lateral sclerosis (ALS) have led to the description of a phenotype referred to as disease-associated microglia, associated with significantly reduced expression of several signature GPCR genes (14). When comparing gene expression in pure microglia from normal-appearing, non-lesional tissue of deceased multiple sclerosis (MS) autopsy cases and tissue of non-pathological brains (**Figure 2** and **Supplementary Table 2**), we found downregulation of *ADGRG1* in both, white and grey matter, while expression of other GPCR genes was not changed.

**Figure 3:**
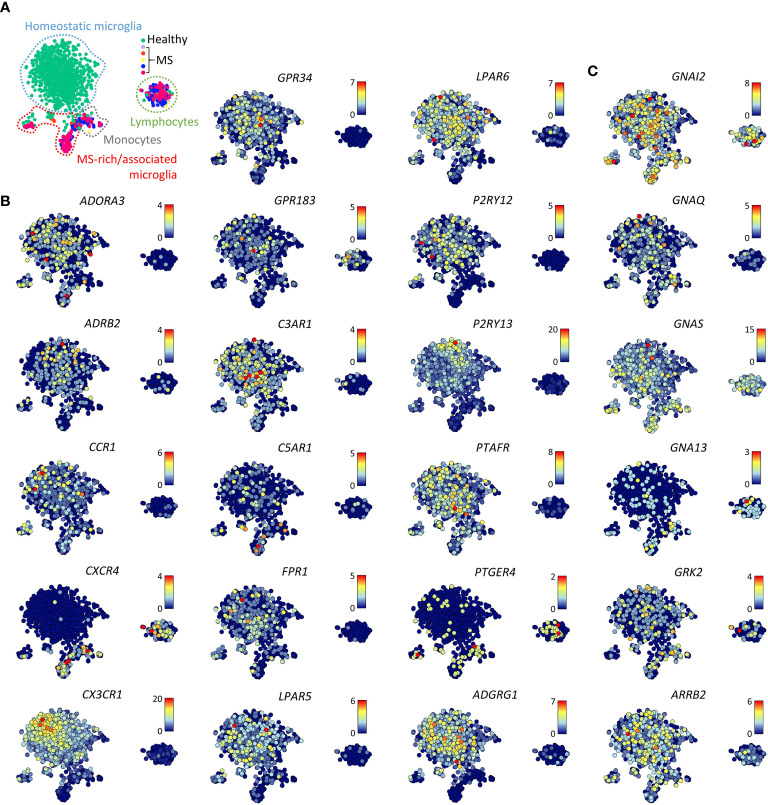
Gene expression of selected GPCRs and GPCR signaling molecules in MS lesions. **(A)** *t*-SNE plot of 1,602 individual cells isolated from five non-pathological brains (healthy) and five brains of patients with early active MS representing with indicated homeostatic microglia, MS- enriched/associated microglia, monocytes, and lymphocytes. **(B, C)** *t*-SNE plots of GPCR **(B)** and GPCR signaling molecule **(C)** genes (15). Color codes represent expression levels.

Single cell RNAseq data of (models of) Alzheimer’s disease, ALS, and MS revealed that pathological reprogramming only occurs in small subsets of disease-associated microglia that coexist with large subsets of homeostatic microglia and small subsets of infiltrating monocytes (14–16). Notably, disease-associated microglia in lesion biopsies of patients with histologically confirmed early active MS pathology showed reduced expression of the core signature genes *CX3CR1*, *GPR34*, *GPR183*, *P2RY12*, *P2RY13*, and *ADGRG1* (15, 26) (**Figures 3A, B**). In contrast, both disease-associated microglia and infiltrating monocytes had upregulated expression of *CXCR4*. In disease-associated microglia in surgically resected material of Alzheimer’s disease patients, GPCR expression was not altered (16).

The late onset of neurodegenerative diseases, such as Alzheimer’s disease, Parkinson’s disease, and ALS, has triggered interest in the effect of aging on microglia gene expression. A study of aged microglia found a lower expression of the microglia signature genes *GPR183*, *P2RY12*, and *P2RY13* (17).

Expression of GPCR signaling molecule genes in the data sets studied here was quite stable. Separation of microglia from their CNS microenvironment or transfer of iMPs into NSG mouse brains only moderately affected signaling molecule gene expression (**Figure 2**). However, white matter microglia more abundantly expressed *GNA13* as compared to grey mater microglia. GPCR signaling molecule genes expression was not altered in normal-appearing or lesional MS microglia from either white or grey matter (**Figure 3C**).

### Expression and MS-Associated Upregulation of CXCR4 by Human White Matter Microglia

**Figure 4:**
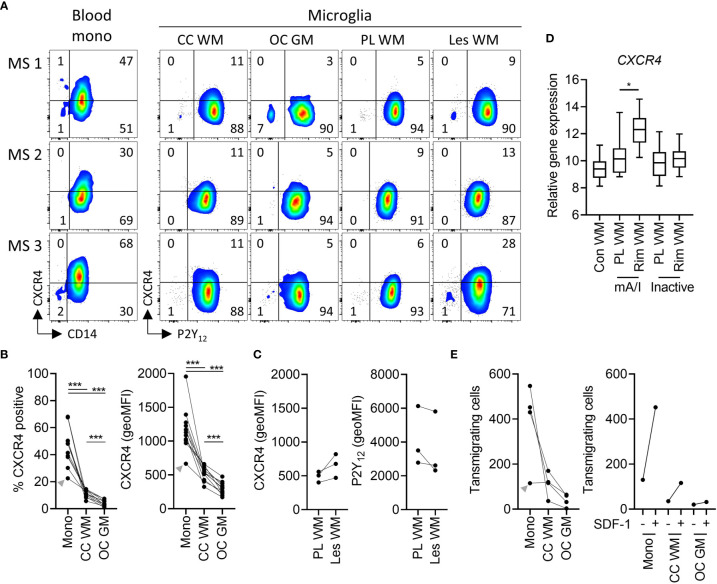
Expression of functional CXCR4 in white and grey matter microglia in health and MS. **(A)** Representative dot plots of CXCR4 expression on paired peripheral CD14^+^ blood monocytes and P2Y_12_^+^ microglia from corpus callosum white matter and occipital cortex grey matter, as well as P2Y_12_^+^ microglia from peri-lesional and lesional subcortical WM of three MS brain donors measured by flow cytometry. **(B)** Quantification of CXCR4 expression (percentage of positive cells and geoMFI) on monocytes, WM microglia, and GM microglia of n=11 brain donors (3 MS, 8 non-MS; Wilcoxon-signed rank test; ***p < 0.0005). **(C)** Quantification of CXCR4 and P2Y_12_ expression on microglia from peri-lesional and lesional WM of three MS brain donors. **(D)** Quantification of tissue gene expression of *CXCR4* in control as well as peri-lesional and lesional WM from mixed active/inactive and inactive MS lesions (18) (Wilcoxon-signed rank test; *p < 0.05). **(E)** Quantification of transwell migration of monocytes, white matter microglia, and grey matter microglia in response to the CXCR4 ligand SDF-1 of n=4 brain donors (left panel). Of note, SDF-1-stimulated transwell migration was higher as compared to spontaneous transwell migration in all three cell types (n=1 brain donor; right panel). A donor with a relatively low expression of CXCR4 on monocytes also showed low monocyte transmigration towards SDF-1 (grey arrowhead). mono, monocytes; CC, corpus callosum; WM, white matter; OC, occipital cortex; GM, grey matter; PL, peri-lesional; Les, lesional; mA/I, mixed active/inactive; geoMFI, geometric mean fluorescence intensity.

The presence and MS-associated upregulation of *CXCR4* expression in microglia is of particular interest since Werner et al. recently showed that CXCR4 distinguishes monocytes from microglia in mice (27). To test whether human microglia express CXCR4, we analyzed freshly isolated cells by flow cytometry, shown here for three donors with MS (**Figure 4A**). Expression was detectable, albeit levels were moderate on white matter microglia and low on grey matter microglia (**Figures 4A, B**). On lesional microglia, we noticed a slightly higher expression of CXCR4 as well as lower expression of P2Y_12_ compared to microglia from subcortical normal-appearing white matter, the latter in line with Zrzavy et al. (26) (**Figures 4A, C**). Whole tissue gene expression microarray analysis of laser-dissected control white matter and white matter MS lesions (18) further confirmed increased expression of *CXCR4* in the rim of mixed active/inactive but not inactive lesions (**Figure 4D**).

To test whether expression of CXCR4 on microglia is functional, we studied transwell migration in response to SDF-1 (CXCL12) (14). SDF-1 binds to CXCR4 and CXCR7, the latter however is not expressed by microglia (**Supplementary Table 2**). Of note SDF-1 stimulated chemotaxis of monocytes, white matter and grey matter microglia at levels corresponding with the presence of CXCR4 (**Figure 4E**). We conclude that CXCR4 expression on human microglia is functional.

## Discussion

GPCRs constitute an important share of the sensome of eukaryotic cells. By analyzing various RNAseq datasets, we here provide a comprehensive overview of their presence in microglia. We identified 17 GPCR genes that are robustly transcribed in adult human microglia, including the homeostatic core genes *CX3CR1*, *GPR34*, *GPR183*, *P2RY12*, *P2RY13*, and *ADGRG1*, but also *ADORA3*, *ADRB2, CCR1*, *C3AR1*, *C5AR1*, *LPAR5*, *LPAR6*, and *PTAFR*. GPCRs genes well expressed in human microglia but hardly found in mouse microglia were *CXCR4*, *FPR1*, and *PTGER4*.

**Figure 5:**
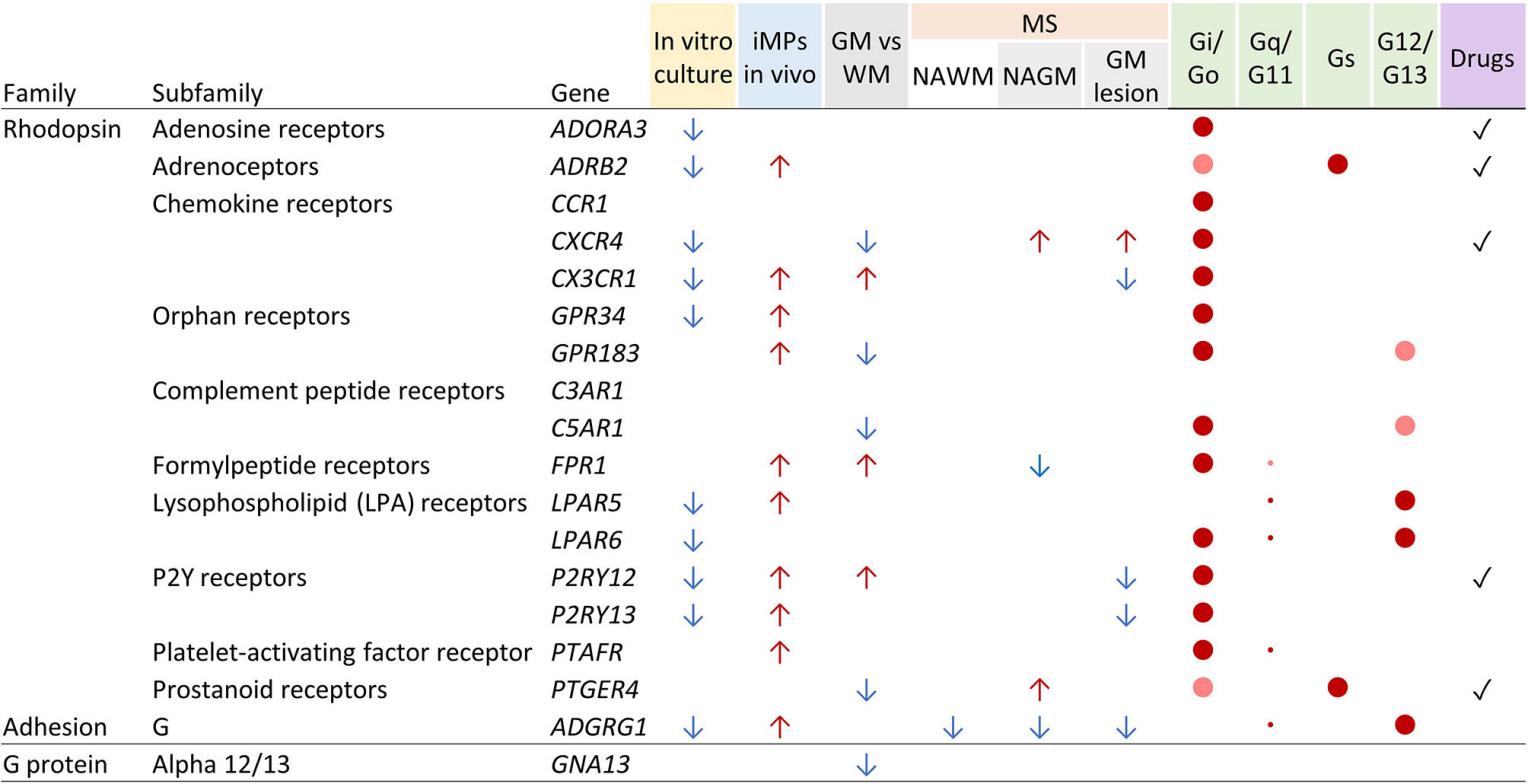
Tabular summary of human microglia GPCR gene regulation, signaling, and drug availability. See Discussion for details. Arrows indicate the direction of regulation. Colored dots indicate prevalence of signaling of GPCRs through the respective G proteins (red = high; light red = medium) and abundance of gene expression of the G proteins in microglia (large dots = high; small dots = low). WM, white matter; GM, grey matter; NAGM, normal-appearing grey matter; NAWM, normal-appearing white matter.

**Figure 5** summarizes the findings of this study. As expected, isolated microglia rapidly lost expression of several GPCRs when cultured *ex vivo*, which limits the value of *in vitro*-expanded primary cells for functional studies. In contrast, iPSC-derived iMPs had a GPCR expression remarkably similar to primary microglia, which further equalized upon transfer into NSG mouse brains, ectopically providing critical human growth factors building the microglia phenotype. Regional diversity of microglia has been suggested (2), and we indeed found more abundant expression of *CXCR4* and *PTGER4* in white matter as compared to grey matter microglia in two independent studies [(4) and Mizee et al., unpublished data].

Disease-associated microglia in mouse models of AD and ALS downregulate expression of various GPCR genes, including *Cx3cr1*, *P2ry12*, *P2ry13*, and *Adgrg1* (14). The data presented here refer to MS, an inflammatory demyelinating and neurodegenerative disease. Bulk primary microglia collected post-mortem from normal-appearing tissue of MS donors showed downregulation of *ADGRG1* (4) in grey and white matter. *ADGRG1* encodes the adhesion family GPCR GPR56 (28), which is more widely expressed in the CNS and has been linked in neuronal precursors with cortical lamination and in oligodendrocyte precursors with proliferation (29). Abundant presence of GPR56 on human microglia (4) and regulation of synaptic refinement through a mouse GPR56 splicing isoform (30) have been reported only recently. Obviously, *ADGRG1* expression not only distinguishes microglia from other macrophages but also fades out in response to minor changes in the microenvironment, stressing its value as indicator of microglia homeostasis. In biopsies of active lesions in early cases of MS, diseases-associated microglia subsets showed the expected general downregulation of GPCR genes associated with microglia homeostasis. Single-cell mass cytometry indeed confirmed the appearance of active MS lesion-enriched clusters with a down-regulated surface expression of P2Y_12_, CX3CR1, and GPR56 (31).

The distribution of the chemokine receptor CXCR4 is of particular interest. CXCR4 binds CXCL12 (SDF-1), which is expressed by the brain vasculature and upregulated in MS lesions (32). Werner et al. recently reported that CXCR4 distinguishes brain-infiltrating monocytes from resident microglia in mice (27). We here demonstrate that human microglia from white and – to a lesser extent – grey matter express CXCR4. Transmigration towards SDF-1 confirmed the relevance of the presence of CXCR4 on microglia. Expression of CXCR4 by both microglia and infiltrating monocytes and upregulation by small subset of MS-associated lesional microglia (15) makes this chemokine receptor, involved in immune cell homeostasis and margination (33), a potentially interesting target for therapeutic intervention. Indeed, *Cxcr4* gene ablation reduced monocyte infiltration and response gene expression in experimental stroke in mice (27).

When testing the expression of genes encoding signaling molecules downstream of GPCRs, in particular Gα proteins, GRKs, and β-arrestin (6), we found robust expression of *GNAI2*, *GNAS*, *GNA13*, *GRK2*, and *ARRB2*. Further, mice, but not human, abundantly expressed *Gna15*. Expression of these genes did not depend on the specific microenvironment of microglia and was found at similar levels in other macrophages. Gα_s_-coupled receptors activate adenylate cyclase, leading to cAMP accumulation. While *GNAS* transcripts were abundant in microglia, only two highly expressed GPCRs primarily couple to this Gα subunit (*ADRB2*, *PTGER4*) suggesting rather limited augmentation of cAMP levels in microglia through GPCR signaling. Gα_i_-coupled receptors inhibit the cAMP-dependent pathway by inhibiting adenylyl cyclase activity. Our data indicate that Gα_i_, the preferred Gα subunit of 12 of the 17 abundant microglia GPCRs, is well expressed in microglia, implying inhibition of cAMP-dependent protein kinase (PKA). Gα_12/13_-coupled receptors activate the small GTPase RhoA. We found *GNA13* abundantly transcribed in white but not grey matter, which explains why initial studies of cortical microglia reported dim expression (10). Finally, Gα_q_-coupled receptors activate phospholipase C to increase intracellular calcium concentration as well as activate protein kinase C, which results in Raf kinase activation of the MAPK pathway. *GNAQ* transcript levels were generally low, suggesting that LPAR5, LPAR6, and GPR56, which can engage Gα_12/13_ as well as Gα_q_, may rather control cell cytoskeleton remodeling and thus regulate microglia migration. Yet, in particular for GPR56, molecular mechanisms additional to G protein signaling may apply (28, 34).

GPCRs are known for their excellent drugability. A survey at DrugBank (https://www.drugbank.ca/) revealed approved drugs against at least five highly expressed microglia GPCRs with indications covering, amongst others, conditions of the lungs (ADORA3, ADRB2), eye (ADRB2), blood (CXCR4, P2Y_12_), heart (P2Y_12_), and uterus (PTGER4). Moreover, rodent models suggest efficacy of CXCR4 targeting in the treatment of stroke and glioma (35). Studying the effects of small molecules, penetrating the blood–brain barrier, on microglia *in vitro* and *in vivo* may disclose novel opportunities for the treatment of brain diseases in which microglia emerge as central players, including neurodevelopmental disorders (e.g., autism), neurodegenerative and -inflammatory conditions (e.g., Alzheimer’s disease and MS), and chronic pain (1).

In summary, we here describe the GPCR repertoire of human microglia based on publicly available bulk and single-cell RNAseq data. GPCRs that belong to the core signature of microglia are abundantly expressed under homeostatic and rapidly downregulated under non-homeostatic conditions, making them interesting models for studying microenvironmental factors that shape microglia identity during brain development and disease. Datasets of microglia from brain donors with neurological diseases only lately became available and require further investigation. This in particular holds true as drugs targeting different highly expressed microglia GPCRs have been developed, implying their potential application for CNS diseases in which microglia figurate.

## Data Availability Statement

The datasets presented in this study can be found in online repositories. The names of the repository/repositories and accession number(s) can be found below: https://www.ncbi.nlm.nih.gov/ (accessible under accession code GEO: GSE52564, GSE98969, GSE99074, GSE108000, GSE111972, GSE124335, GSE128855, GSE134707, GSE139194), https://www.synapse.org/#!Synapse:syn21438358 and http://shiny.maths.usyd.edu.au/Ellis/MicrogliaPlots/.

## Ethics Statement

Written informed consent was obtained from the individual(s) for the publication of any potentially identifiable images or data included in this article.

## Author Contributions

C-CH and RS extracted and analyzed data. C-CH, RS, MP, JS, IH, and JH designed research and interpreted results. C-CH and JH wrote the paper. All authors contributed to the article and approved the submitted version.

## Funding

The German Research Foundation (FOR 2149 – JH), the Berta-Ottenstein-Programme for Clinical Scientists (RS), the MS Research Foundation (MS 13-830 – IH/JH), and the Nationaal MS Fonds (OZ2018-003 – JS) funded this research.

## Conflict of Interest

The authors declare that the research was conducted in the absence of any commercial or financial relationships that could be construed as a potential conflict of interest.

## References

[1] Salter MW Stevens B. Microglia Emerge as Central Players in Brain Disease. Nat Med (2017) 23:1018–27. 10.1038/nm.4397 28886007

[2] Prinz M Jung S Priller J. Microglia Biology: One Century of Evolving Concepts. Cell (2019) 179:292–311. 10.1016/j.cell.2019.08.053 31585077

[3] Crotti A Ransohoff RM. Microglial Physiology and Pathophysiology: Insights From Genome-wide Transcriptional Profiling. Immunity (2016) 44:505–15. 10.1016/j.immuni.2016.02.013 26982357

[4] van der Poel M Ulas T Mizee MR Hsiao CC Miedema SSM Adelia. Transcriptional Profiling of Human Microglia Reveals Grey–White Matter Heterogeneity and Multiple Sclerosis-Associated Changes. Nat Commun (2019) 10:1139. 10.1038/s41467-019-08976-7 30867424 PMC6416318

[5] Hauser AS Attwood MM Rask-Andersen M Schiöth HB Gloriam DE. Trends in GPCR Drug Discovery: New Agents, Targets and Indications. Nat Rev Drug Discov (2017) 16:829–42. 10.1038/nrd.2017.178 PMC6882681 29075003

[6] Pierce KL Premont RT Lefkowitz RJ. Seven-Transmembrane Receptors. Nat Rev Mol Cell Biol (2002) 3:639–50. 10.1038/nrm908 12209124

[7] Li Q Barres BA. Microglia and Macrophages in Brain Homeostasis and Disease. Nat Rev Immunol (2018) 18:225–42. 10.1038/nri.2017.125 29151590

[8] Geirsdottir L David E Keren-Shaul H Weiner A Bohlen SC Neuber J. Cross-Species Single-Cell Analysis Reveals Divergence of the Primate Microglia Program. Cell (2019) 179:1609–22.e16. 10.1016/j.cell.2019.11.010 31835035

[9] Galatro TF Holtman IR Lerario AM Vainchtein ID Brouwer N Sola PR. Transcriptomic Analysis of Purified Human Cortical Microglia Reveals Age-Associated Changes. Nat Neurosci (2017) 20:1162–71. 10.1038/nn.4597 28671693

[10] Gosselin D Skola D Coufal NG Holtman IR Schlachetzki JCM Sajti E. An Environment-Dependent Transcriptional Network Specifies Human Microglia Identity. Science (2017) 356:1248–59. 10.1126/science.aal3222 PMC5858585 28546318

[11] Zhang Y Chen K Sloan SA Bennett ML Scholze AR O’Keeffe S. An RNA-sequencing Transcriptome and Splicing Database of Glia, Neurons, and Vascular Cells of the Cerebral Cortex. J Neurosci (2014) 34:11929–47. 10.1523/JNEUROSCI.1860-14.2014 PMC4152602 25186741

[12] Van Hove H Martens L Scheyltjens I De Vlaminck K Pombo Antunes AR De Prijck S. A Single-Cell Atlas of Mouse Brain Macrophages Reveals Unique Transcriptional Identities Shaped by Ontogeny and Tissue Environment. Nat Neurosci (2019) 22:1021–35. 10.1038/s41593-019-0393-4 31061494

[13] Svoboda DS Barrasa MI Shu J Rietjens R Zhang S Mitalipova M. Human iPSC-derived Microglia Assume a Primary Microglia-Like State After Transplantation Into the Neonatal Mouse Brain. Proc Natl Acad Sci USA (2019) 116:25293–303. 10.1073/pnas.1913541116 PMC6911218 31772018

[14] Keren-Shaul H Spinrad A Weiner A Matcovitch-Natan O Dvir-Szternfeld R Ulland TK. A Unique Microglia Type Associated With Restricting Development of Alzheimer’s Disease. Cell (2017) 169:1276–1290.e17. 10.1016/j.cell.2017.05.018 28602351

[15] Masuda T Sankowski R Staszewski O Böttcher C Amann L Sagar. Spatial and Temporal Heterogeneity of Mouse and Human Microglia At Single-Cell Resolution. Nature (2019) 566:388–92. 10.1038/s41586-019-0924-x 30760929

[16] Olah M Menon V Habib N Taga MF Ma Y Yung CJ. Single Cell RNA Sequencing of Human Microglia Uncovers a Subset Associated With Alzheimer’s Disease. Nat Commun (2020) 11:6129. 10.1038/s41467-020-19737-2 33257666 PMC7704703

[17] Olah M Patrick E Villani AC Xu J White CC Ryan KJ. A Transcriptomic Atlas of Aged Human Microglia. Nat Commun (2018) 9:539. 10.1038/s41467-018-02926-5 29416036 PMC5803269

[18] Hendrickx DAE van Scheppingen J van der Poel M Bossers K Schuurman KG van Eden CG. Gene Expression Profiling of Multiple Sclerosis Pathology Identifies Early Patterns of Demyelination Surrounding Chronic Active Lesions. Front Immunol (2017) 8:1810. 10.3389/fimmu.2017.01810 29312322 PMC5742619

[19] Mizee MR Miedema SSM van der Poel M Adelia Schuurman KG van Strien ME. Isolation of Primary Microglia From the Human Post-Mortem Brain: Effects of Ante- and Post-Mortem Variables. Acta Neuropathol Commun (2017) 5:16. 10.1186/s40478-017-0418-8 28212663 PMC5316206

[20] Alexander SPH Christopoulos A Davenport AP Kelly E Mathie A Peters JA. The CONCISE Guide TO Pharmacology 2019/20: G Protein-Coupled Receptors. Br J Pharmacol (2019) 176:S21–141. 10.1111/bph.14748 31710717 PMC6844580

[21] Schiöth HB Lagerström MC. Structural Diversity of G Proteincoupled Receptors and Significance for Drug Discovery. Nat Rev Drug Discov (2008) 7:339–57. 10.1038/nrd2518 18382464

[22] Hamann J Koning N Pouwels W Ulfman LH van Eijk M Stacey M. EMR1, the Human Homolog of F4/80, is an Eosinophil-Specific Receptor. Eur J Immunol (2007) 37:2797–802. 10.1002/eji.200737553 17823986

[23] Lin HH Hsiao CC Pabst C Hébert J Schöneberg T Hamann J. Adhesion GPCRs in Regulating Immune Responses and Inflammation. Adv Immunol (2017) 136:163–201. 10.1016/bs.ai.2017.05.005 28950945

[24] Visser L De Vos AF Hamann J Melief MJ Van Meurs M Van Lier RAW. Expression of the EGF-TM7 Receptor CD97 and its Ligand CD55 (DAF) in Multiple Sclerosis. J Neuroimmunol (2002) 132:156–63. 10.1016/S0165-5728(02)00306-5 12417446

[25] Hsiao CC Van Der Poel M Van Ham TJ Hamann J. Macrophages do Not Express the Phagocytic Receptor BAI1/ADGRB1. Front Immunol (2019) 10:962. 10.3389/fimmu.2019.00962 31130954 PMC6509540

[26] Zrzavy T Hametner S Wimmer I Butovsky O Weiner HL Lassmann H. Loss of “Homeostatic” Microglia and Patterns of Their Activation in Active Multiple Sclerosis. Brain (2017) 140:1900–13. 10.1093/brain/awx113 PMC6057548 28541408

[27] Werner Y Mass E Ashok Kumar P Ulas T Händler K Horne A. Cxcr4 Distinguishes HSC-derived Monocytes From Microglia and Reveals Monocyte Immune Responses to Experimental Stroke. Nat Neurosci (2020) 23:351–62. 10.1038/s41593-020-0585-y PMC7523735 32042176

[28] Hamann J Aust G Araç D Engel FB Formstone C Fredriksson R. International Union of Basic and Clinical Pharmacology. XCIV. Adhesion G Protein-Coupled Receptors. Pharmacol Rev (2015) 67:338–67. 10.1124/pr.114.009647 PMC4394687 25713288

[29] Langenhan T Piao X Monk KR. Adhesion G Protein-Coupled Receptors in Nervous System Development and Disease. Nat Rev Neurosci (2016) 17:550–61. 10.1038/nrn.2016.86 27466150

[30] Li T Chiou B Gilman CK Luo R Koshi T Yu D. A Splicing Isoform of GPR56 Mediates Microglial Synaptic Refinement Via Phosphatidylserine Binding. EMBO J (2020) 39:e104136. 10.15252/embj.2019104136 32452062 PMC7429740

[31] Böttcher C Van Der Poel M Fernández-Zapata C Schlickeiser S Leman JKH Hsiao CC. Single-Cell Mass Cytometry Reveals Complex Myeloid Cell Composition in Active Lesions of Progressive Multiple Sclerosis. Acta Neuropathol Commun (2020) 8:136. 10.1186/s40478-020-01010-8 32811567 PMC7437178

[32] Krumbholz M Theil D Cepok S Hemmer B Kivisäkk P Ransohoff RM. Chemokines in Multiple Sclerosis: CXCL12 and CXCL13 Up-Regulation is Differentially Linked to CNS Immune Cell Recruitment. Brain (2006) 129:200–11. 10.1093/brain/awh680 16280350

[33] Nagasawa T. CXC Chemokine Ligand 12 (CXCL12) and its Receptor CXCR4. J Mol Med (2014) 92:433–9. 10.1007/s00109-014-1123-8 24722947

[34] Langenhan T Aust G Hamann J. Sticky Signaling - Adhesion Class G Protein-Coupled Receptors Take the Stage. Sci Signal (2013) 6:re3. 10.1126/scisignal.2003825 23695165

[35] Tahirovic YA Pelly S Jecs E Miller EJ Sharma SK Liotta DC. Small Molecule and Peptide-Based CXCR4 Modulators as Therapeutic Agents. A Patent Review for the Period From 2010 to 2018. Expert Opin Ther Pat (2020) 30:87–101. 10.1080/13543776.2020.1707186 31854208

