# Supplementary figures and images for: GPCRomics of Homeostatic and Disease-Associated Human Microglia
Source: Front Immunol. 2021 May 14;12:674189. doi: 10.3389/fimmu.2021.674189 (PMC8160299; doi:10.3389/fimmu.2021.674189)

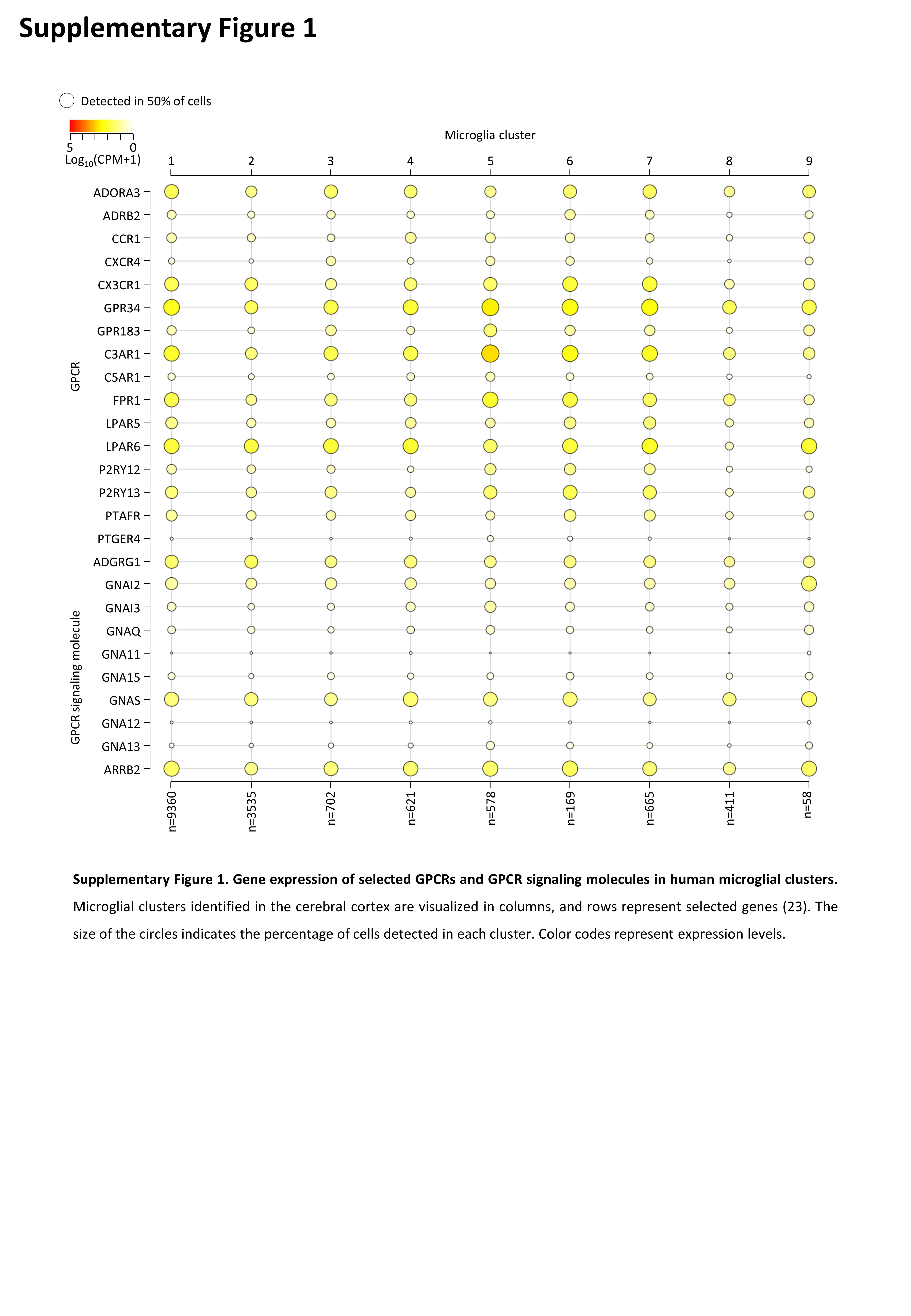

Supplement: Supplementary file 1 [file Image_1.jpeg]
